# Designing Conductive‐Bridge Phase‐Change Memory to Enable Ultralow Programming Power

**DOI:** 10.1002/advs.202103478

**Published:** 2022-01-14

**Authors:** Zhe Yang, Bowen Li, Jiang‐Jing Wang, Xu‐Dong Wang, Meng Xu, Hao Tong, Xiaomin Cheng, Lu Lu, Chunlin Jia, Ming Xu, Xiangshui Miao, Wei Zhang, En Ma

**Affiliations:** ^1^ Wuhan National Laboratory for Optoelectronics School of Optical and Electronic Information Huazhong University of Science and Technology Wuhan 430074 China; ^2^ Center for Alloy Innovation and Design (CAID) State Key Laboratory for Mechanical Behavior of Materials Xi'an Jiaotong University Xi'an 710049 China; ^3^ The School of Microelectronics State Key Laboratory for Mechanical Behavior of Materials Xi'an Jiaotong University Xi'an 710049 China

**Keywords:** chalcogenide glass, low‐power devices, materials design, non‐volatile memory, phase‐change memory

## Abstract

Phase‐change material (PCM) devices are one of the most mature nonvolatile memories. However, their high power consumption remains a bottleneck problem limiting the data storage density. One may drastically reduce the programming power by patterning the PCM volume down to nanometer scale, but that route incurs a stiff penalty from the tremendous cost associated with the complex nanofabrication protocols required. Instead, here a materials solution to resolve this dilemma is offered. The authors work with memory cells of conventional dimensions, but design/exploit a PCM alloy that decomposes into a heterogeneous network of nanoscale crystalline domains intermixed with amorphous ones. The idea is to confine the subsequent phase‐change switching in the interface region of the crystalline nanodomain with its amorphous surrounding, forming/breaking “nano‐bridges” that link up the crystalline domains into a conductive path. This conductive‐bridge switching mechanism thus only involves nanometer‐scale volume in programming, despite of the large areas in contact with the electrodes. The pore‐like devices based on spontaneously phase‐separated Ge_13_Sb_71_O_16_ alloy enable a record‐low programming energy, down to a few tens of femtojoule. The new PCM/fabrication is fully compatible with the current 3D integration technology, adding no expenses or difficulty in processing.

## Graphical abstract

The high‐power consumption in phase‐change memory remains a bottleneck problem limiting data storage density. Here, a conductive‐bridge switching mechanism with a self‐decomposed Ge_13_Sb_71_O_16_ alloy is demonstrated, which results in a heterogeneous network of nanoscale amorphous/crystalline domains, confining the phase‐change switch in a “nano‐bridge” at the interface between amorphous and crystalline domains. A record‐low programming energy is achieved in conventional‐sized devices.

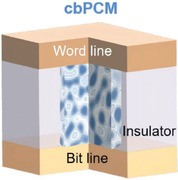

## 1 Introduction

Artificial intelligence (AI) and other data‐intensive technologies are pushing forward the frontiers of non‐volatile memory and neuro‐inspired computing devices. Correspondingly, the requirements on the materials implementing these functions have also escalated, demanding increasingly fast switching speed, long‐term data retention, low power consumption, high cycling endurance, low resistance drift, and efficient emulations of artificial neural network. Chalcogenide phase‐change materials (PCM), based on the Ge_2_Sb_2_Te_5_ (GST) alloy currently used in commercial memory products, are one of the leading material candidates^[^ ^1^, ^2^, ^3^, ^4^, ^5^, ^6^, ^7^, ^8^, ^9^, ^10^ ^]^ with properties being systematically tailored toward one (or more) of the targeted directions listed above.^[^ ^2^, ^11^ ^]^ For instance, the recently designed Sc_0.2_Sb_2_Te_3_ alloy with ultrafast nucleation rate brings the programming time down to hundreds of picoseconds, extending the memory‐oriented application of PCM toward non‐volatile cache‐type memories.^[^ ^12^, ^13^ ^]^ Another example is the new concept of phase‐change heterostructure (PCH), employing TiTe_2_/Sb_2_Te_3_ as confinement/PCM nanolayers. The PCH enables memory programming with ultralow drift and noise, potentially suitable for neuro‐inspired computing operations of high accuracy and consistency.^[^ ^14^, ^15^ ^]^ In the following, we aim to push the envelope for yet another key attribute, that is, energy consumption needed to accomplish memory switching. This is important for storage‐oriented applications, particularly in compact 3D stacked arrays where heat dissipation has been a nagging problem. In fact, the current 3D XPoint PCM product is limited to a few stackable layers, because otherwise the large amount of heat generated during the RESET process would degrade the device quickly. Minimizing the power consumption for energy‐efficient memories therefore remains a major challenge.

The SET and RESET operations in phase‐change memory devices are accomplished by crystallization and amorphization of the PCM under external electrical pulses through joule heating. The large contrast in electrical resistance between the amorphous phase—high resistance state (HRS “0”) and the crystalline phase—low resistance state (LRS “1”) of PCM is used to encode digital information. The major power cost in devices resides with the RESET operation, in which the crystalline phase needs to be melted first at temperature *T*_m_ ≈ 900 K, and then rapidly quenched to form the amorphous phase. Typical PCM devices require tens of picojoule to nanojoule and a large current density of >1 MA cm^−2^ for each RESET operation.^[^ ^16^ ^]^ To reduce the RESET energy *E*_RESET_, various approaches have been developed, including materials optimization and design,^[^ ^12^, ^17^, ^18^, ^19^, ^20^, ^21^ ^]^ interface and electrode engineering,^[^ ^22^, ^23^, ^24^, ^25^ ^]^ nanowires,^[^ ^26^, ^27^ ^]^ nanocrystals,^[^ ^28^, ^29^ ^]^ as well as superlattices.^[^ ^30^, ^31^, ^32^, ^33^ ^]^ Yet the power consumption remained on the order of multiple picojoules or the RESET current ranged from tens to hundreds of µA. An extreme case was reported in ref. [34], where nanoscale gaps were created in carbon nanotubes (CNT), and then filled with ≈10 nm thick GST films. An ultralow *E*_RESET_ ≈ 100 fJ was achieved in this device setup, with a RESET current ≈5 µA, as the contact area between the CNT electrodes (diameter ≈ 3 nm) and GST, that is, the switching (programming) volume, is made extremely small. The energy consumption is further improved to ≈80 fJ by using self‐aligned GST nanowires and CNT electrodes (diameter ≈ 1.7 nm), with a RESET current ≈1.6 µA.^[^ ^35^ ^]^ However, such a nanowire/tube route requires delicate nanofabrication protocol, posing formidable challenges for the mass production of PCM devices and chips. Therefore, there is a pressing need for a practical materials solution toward processing‐friendly low‐power devices.^[^ ^36^ ^]^

**Figure 1:**
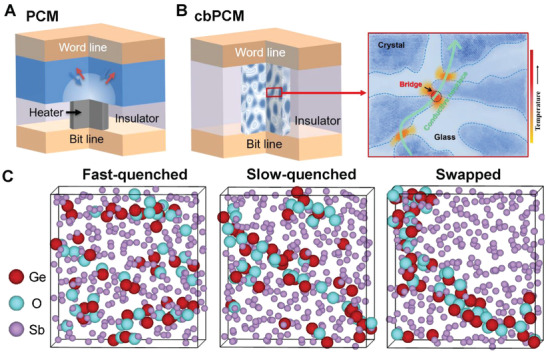
Design concept. A) Sketch of conventional mushroom‐type memory. B) Concept of conductive‐bridge phase‐change memory (cbPCM), showing the programmable layer with necklace‐like network comprised of crystalline nanodomains intertwined with amorphous domains (left panel), and a potential conductive bridge linking nearby crystalline nanodomains for charge transport (close‐up view in the right panel). Phase change is expected to occur mostly at the narrow connection gaps (open or close the “bridge”). C) The DFMD snapshots of the Ge‐Sb‐O model after fast and slow quenching, and the swapped model. The Ge, Sb, and O atoms are rendered with red, purple, and cyan spheres. The size of Ge and O atoms is enlarged for a clear view of the co‐segregation of Ge and O. The degree of local enrichment of Ge and O in the amorphous state increased upon slow quenching.

All the approaches so far follow a direct scaling between the power consumption and the size of the data storage device. This correlation also holds for PCM memory cells. As seen in the mushroom‐shape geometry shown in **Figure** **1**A, the programming volume is determined by the contact area of the PCM with the bottom electrode. The smaller the diameter of the bottom electrode, the lower the power consumed in the switching operation. So far, the size of the bottom electrode is down to few tens of nm,^[^ ^25^ ^]^ and further device miniaturization is still being pursued. In this work, we present a materials design strategy to break the proportional dependence of power consumption on device size, leading to an ultralow *E*_RESET_ of tens of fJ in a normal‐sized device (250 nm hole diameter). The new alloy scheme enables a drastic energy reduction, by more than four orders of magnitude, with respect to GST devices in the same device geometry. The alloy can be synthesized through sputter deposition with no added fabrication procedure, which provides an alternative approach for high‐density integration of PCM cells.

## 2 Results

### 2.1 The Concept of Conductive‐Bridge Phase‐Change Memory

The essence of our idea is to create inside the PCM alloy a conductive channel with the dimension of a few nanometers, such that the switching volume is minimized and the current path can be easily open and shut. To this end, our conductive channel is made of low‐resistance and low‐*T*_c_ PCM regions, that is, crystalline nanodomains (CNDs) chained together, which are surrounded by high‐resistance amorphous regions. These CNDs are nearly‐interconnected, separated only by minute amorphous gaps. The amorphous‐crystalline phase change during cyclic switching is then executed merely in the left‐over gaps in between the CNDs. In other words, the electrical resistance of the current path is controlled by the tiny bridges linking up the CNDs. This scheme makes use of joule‐heating induced phase transition at edges, which was demonstrated possible in a nanofabrication‐patterned (and doped‐) GST memory cell (reducing the RESET current to 20 µA).^[^ ^29^ ^]^ We aim to i) design a PCM alloy to spontaneously generate nano‐sized conductive channel in a normal‐sized cell without lithography patterning, ii) minimize the effective switching volume to only one conductive channel, and as such iii) reduce the RESET current and power consumption to a few µA and tens of fJ.

To make this advance possible, the PCM we desire is an alloy that spontaneously decomposes into a network of nanodomains having spatially varied chemical composition and thermal and electrical resistance. As schematically depicted in Figure 1B, the electric current and the resulting joule heat are likely to be concentrated to form a conductive necklace across many CNDs, by converting the missing link (red region) into conductive bridge. For this conductive‐bridge PCM (cbPCM) scheme to work, the alloy selection needs to meet the following criteria. First, the base PCM should be of the growth‐type, such that the phase change is in the form of expansion/shrinkage at the crystalline‐amorphous interface. Stochastic and undesired nucleation inside the amorphous domains is to be suppressed. Second, a small addition of alloying element should lead to strong phase separation with smooth interfaces under external stimulus such as heating or electrical pulses, leading spontaneously to the desired necklace‐like network microstructure. Third, one set of the domains should be highly resistive to both electrical currents and heat flow, staying robust in amorphous state (with a high *T*_c_) that remains nearly unchanged during service. This necessitates an alloying element as a strong stabilizer for the amorphous phase. Fourth, the other set of domains should have low *T*_c_ and *T*_m_, offering a low barrier for phase transition. This facilitates the back‐and‐forth expansion/retraction of the crystal boundary around these CNDs, serving the function of a “conductive bridge” that can be “open” or “shut” during cyclic programming. Fifth, the new material should be compatible with the current PCM and semiconductor technology, to keep the fabrication practical for mass production.

### 2.2 Design and Synthesis of Heterogeneous Ge‐Sb‐O Alloys

In the classical PCM Ge‐Sb‐Te ternary diagram, two well‐studied growth‐type PCM families are located around the compositions Sb_2_Te and Sb.^[^ ^1^ ^]^ Alloys based on them have enhanced amorphous stability, and the extensively used materials are Ag_4_In_3_Sb_67_Te_26_ and Ge_15_Sb_85_.^[^ ^37^, ^38^, ^39^, ^40^ ^]^ Here we choose the latter, because Sb has a much lower *T*_c_ and high growth rate,^[^ ^41^, ^42^, ^43^ ^]^ and Ge‐Sb alloys are known to be prone to phase separation after many cycles.^[^ ^44^, ^45^ ^]^ As for the alloying element that further increases the driving force for chemical decomposition, a possible candidate is S, which was reported to induce molecular‐scale phase separation in the Ge‐Sb glass.^[^ ^46^ ^]^ We settled on its homologue oxygen (O). This selection of Ge‐Sb‐O is based on the following indicators.

First, our density functional theory (DFT) calculation shows a difference in the energy of formation, *E*_form_, which is −2.09 and −1.85 eV per atom for Ge‐O and Sb‐O crystals, respectively. The Ge‐O chemical bonds are also quantitatively more robust than Sb‐O, as determined by the crystal orbital Hamilton population^[^ ^47^ ^]^ analysis (Figure S1, Supporting Information). We therefore conjecture that upon heating of amorphous Ge‐Sb‐O, domains enriched in Ge and O would be created, together with Sb‐enriched ones. Second, Ge‐O is known to form very good glasses with crystallization temperature above 950 K,^[^ ^48^ ^]^ which is even higher than the melting temperature of Sb and GST (≈900 K). The third and most direct justification of our alloy selection is that our DFT‐based molecular dynamics (DFMD) simulations indicate a clear trend of phase separation in Ge‐Sb‐O glass. To see this, we constructed a Ge‐Sb‐O model with 36 Ge, 288 Sb, and 36 O atoms, randomized the model at 3000 K for 10 ps to remove any possible atomic correlations, and then quenched it from 1000 K down to 0 K in 5 and 100 ps (denoted as the fast‐quenched and slow‐quenched model). At 600 K, we tentatively swapped the position of some Ge and O atoms with Sb atoms in the Sb‐poor regions of the latter model, and equilibrated the new model for 30 ps and then quenched it down to 0 K in 60 ps (denoted as the swapped model). As shown in Figure 1C, Ge atoms tend to form bonds with O atoms, and most of Ge‐O bonds remain robust during the quenching process. The Ge and O atoms have a clear tendency to cluster together in the slow‐quenched model than the fast‐quenched one, and the former is ≈36 meV per atom lower in energy than the latter. Upon further clustering the Ge and O atoms in the swapped model, the energy difference with respect to the fast‐quenched model is enlarged to ≈41 meV per atom (as a benchmark for comparison, the energy difference between melt‐quenched and aged models of amorphous GeTe is about ≈8 meV per atom^[^ ^49^ ^]^). This indicates a strong tendency toward phase separation into a Ge‐O stabilized glass, and a Sb‐enriched one that is prone to crystallization (i.e., the formation of CNDs) and easy to switch, all inside the amorphous alloy film. As such, all the materials selection criteria in the preceding paragraph are satisfied.

**Figure 2:**
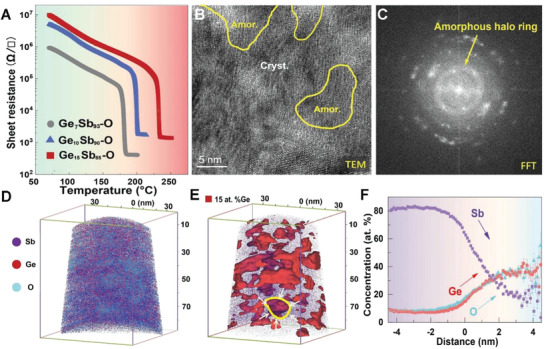
Ge‐Sb‐O alloy thin films. A) Resistance–temperature curves of three Ge‐Sb‐O alloys upon heating. The less the Sb content, the higher the crystallization temperature. The Ge_15_Sb_85_‐O thin film was annealed at 200 °C for 30 min for transmission electron microscope (TEM) and atom probe tomography (APT) characterization. The composition is determined to be Ge_13_Sb_71_O_16_ (GSO) using APT. B) A high‐resolution TEM image of the annealed GSO thin film and C) the corresponding Fast Fourier Transform (FFT) pattern, showing the mixture of amorphous and crystalline domains. D) An overall 3D APT reconstruction of a GSO needle in an 87 × 74 × 71.5 nm^3^ volume, showing an inhomogeneous distribution of Sb (purple), Ge (red), and O (cyan) atoms. E) Iso‐concentration surfaces (in red) of 15 at% Ge. The surface marked by the yellow circle was used for the proximity histogram concentration profiles in (F). F) Approaching the interior region of the red cluster pointed by white arrows, the concentration of Sb changes from ≈80 to ≈20 at% across an interfacial region 2 to 4 nm in width, while that of Ge and O increases sharply.

To see if this new O‐doped PCM works, we synthesized Ge‐Sb‐O thin films ≈100 nm in thickness, with varied compositions from Ge*_x_* Sb_100 −_ *_x_* targets with *x* = 7, 10, and 15, using magnetron sputtering in a mixed argon and oxygen pressure of 0.5 Pa. Note that for most of PCM applications, oxidation needs to be avoided to guard against undesired changes in crystallization performance. Yet, for the applications that require high thermal stability and small density contrast for switching, oxygen doping was also used.^[^ ^50^, ^51^, ^52^, ^53^ ^]^ Previous optical experiments demonstrated that Ge‐Sb maintains the growth‐dominant crystallization mechanism as long as the O alloyed into the system is less than 16%, precluding that undesired nucleation upon device cycling.^[^ ^54^, ^55^ ^]^ Resistance measurements were conducted via four‐probe method in a vacuum chamber for the three Ge‐Sb‐O thin films. As depicted in **Figure** **2**A, the sheet resistances of as‐deposited amorphous films are high at room temperature, and decrease linearly with a typical semiconductor behavior as temperature increases at a rate of 5 K min^−1^. The resistance drops abruptly by two orders of magnitude in the temperature range of 180–230 °C, where crystallization occurs. The lower the Sb content, the better the amorphous stability. These transport profiles show that the crystallization and electrical properties of the thin films are sensitive to chemical composition of Ge‐Sb‐O.

### 2.3 Heterogeneous Network Achieved Under Thermal or Electrical Stimuli

We subjected the Ge‐Sb‐O alloy having the highest stability to thermal annealing, to see if the phase‐separating microstructure would evolve in the way we desire. The thin film, covered by an ≈10 nm electron‐transparent ZnS‐SiO_2_ capping layer (to prevent further oxidation), was annealed at 200 °C for 30 min in a high vacuum furnace. As seen under the transmission electron microscope (TEM), the microstructure is found to be more inhomogeneous than the as‐sputtered case, as we conjectured. It now consists of almost‐interconnected domains of partially crystallized regions coexisting with amorphous regions, as observed in the TEM image in Figure 2B and further confirmed via Fast Fourier Transform (FFT) analysis of the TEM image of selected areas (Figure 2C).

The chemical composition of the thin film is quantitatively determined using atom probe tomography (APT). Overall, the alloy has the composition Ge_13_Sb_71_O_16_ (referred to as GSO hereafter). The variation in local chemical composition is clearly revealed by APT. Figure 2D shows a 3D reconstruction of the GSO specimen, in which Ge, Sb, and O atoms are in red, purple, and cyan (each dot denotes an atom). The heterogeneity in elemental distribution is clearly observed. The complex decomposed network is further mapped out by highlighting the iso‐composition surfaces of 15 at% Ge (red surfaces) in Figure 2E. One such surface is analyzed in detail, shown in Figure 2F, which shows a strong compositional variation from ≈82 at% Sb to only ≈24 at% Sb, moving inward in the direction of the white arrows in Figure 2E. The concentration of O atoms shows a strong correlation with Ge atoms, as expected from the phase separation tendency predicted in our DFMD model of the Ge‐Sb‐O glass. It has been established that crystalline PCMs are stabilized by metavalent bonding (MVB),^[^ ^56^, ^57^, ^58^ ^]^ while covalent bonding prevails in amorphous PCMs. The bonding difference between the two phases is evidenced by their distinct dielectric functions,^[^ ^59^ ^]^ and be revealed in direct measurements of bond rupture events using APT.^[^ ^60^, ^61^, ^62^ ^]^ Our experiments show that the average probability of multiple bond rupture events (e.g., several bonds are broken simultaneously, which is the feature of MVB in crystalline PCMs) is ≈48.8% in Sb‐rich regions, but is only ≈24.4% in the Sb‐poor regions, indicating that the GSO thin film is indeed a heterogeneous network of crystalline and amorphous nanodomains upon heating. Notably, the elemental concentration changes continuously but rapidly at the interface, from Sb‐rich to Sb‐poor across a distance of only ≈2 nm. In other words, the GSO alloy is indeed a suitable precursor for the desirable cbPCM microstructure depicted in Figure 1B.

**Figure 3:**
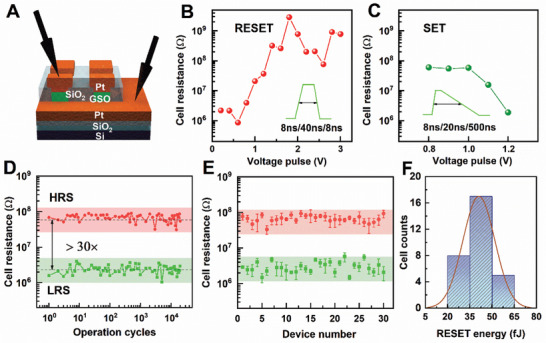
Electrical performance of the GSO device. A) Schematic of the experimental setup for electrical operations of cbPCM devices in a probe station. Resistance–voltage (*R*–*V*) characteristics of the GSO‐*D*_250_ device, B) RESET and C) SET. The full width at half maximum of voltage pulses was used for estimating the power consumption. D) Cycling programming for one typical GSO‐*D*_250_ memory cell. E) Inter‐cell electrical testing. Each cell was programmed over 100 RESET‐SET cycles to obtain an average. F) Statistics of RESET energy distribution for 30 independent memory cells.

From the observations above, the GSO in devices would also undergo “microstructure patterning” into heterogeneous network, if electrical stimuli are used in lieu of thermal annealing. In fact, to set the stage for cbPCM, a necessary step is to accomplish the self‐patterning process using voltage/current, to establish the desired microstructure network needed for subsequent phase switch. We fabricated pore‐like devices with a hole diameter of 250, 500, 750, and 1000 nm, denoted as *D*_250_, *D*_500_, *D*_750_, and *D*_1000_, and filled the pores with sputter‐deposited GSO, as sketched in **Figure** **3**A. A GST‐based device using the same *D*_250_ setup was also prepared, to facilitate a direct comparison. The as‐fabricated device is highly resistive with a cell resistance value above 10^10^ Ω. Cross‐sectional TEM of as‐deposited GSO film shows that it is in a fully amorphous state but with obvious chemical segregation into Sb‐rich and Sb‐poor domains (Figure S2, Supporting Information); this partial network patterning is enhanced by further decomposition during the device fabrication process as a result of the unavoidable rise in temperature. The patterning is subsequently completed by crystallizing the Sb‐rich domains via a couple of 100 ns SET current pulses with an increasing amplitude up to 8 µA. This accomplishes the first SET operation, and the cell resistance turns into LRS with ≈2 × 10^6^ Ω. The device is then ready for subsequent cbPCM memory programming. One caveat to keep in mind here is to avoid too strong SET pulses, because the crystalline fractions could get so high that the cell resistance drops down to kΩ level, making the subsequent RESET operations too difficult (requiring much larger currents). The amplitude of RESET pulses should stay low as well, to avoid large morphological change of the heterogeneous network, for example, large *U*_RESET_ (over 2 eV in Figure 3B) could lead to undesired crystallization.

### 2.4 Ultralow Programming Power for RESET Operations

To investigate the RESET process and find the optimal operation voltage in the GSO‐*D*_250_ device, we applied a series of voltage pulses with the same duration of 48 ns but increasing amplitude up to 3.0 V, see Figure 3B. This results in a gradual increase in cell resistance from LRS (≈10^6^–10^7^ Ω) to HRS (≈10^8^ Ω and above). To SET the GST‐*D*_250_ device into LRS (Figure 3C), voltage pulses with long decay width are needed, while much shorter current pulses, for example, 103 ns, are already sufficient for SET operations (Figure S3, Supporting Information). The resistance window is found to be rather wide, with at least one order of magnitude difference in resistance. It is also robust over time, as shown in Figure S4, Supporting Information, where the measured drift coefficient *ν* is as low as ≈0.003 for LRS and ≈0.014 for HRS. The latter value is much lower than that in GST devices (≈0.11).^[^ ^63^ ^]^ This can be attributed to the fact that the heterogeneous network in our GSO devices corresponds to a well‐aged state, consisting of separated Sb‐rich CNDs (with Sb concentration >80%, Figure 2F) and GeO‐rich glass domains that do not undergo much structural relaxation with time. Aging effects are possible in the switchable edges, where the Sb concentration is lower than 80%, but the volume fraction of these regions is very limited. As such, our GSO devices are not subject to obvious drift in cell resistance (Figure S4, Supporting Information).

Next, we evaluate the cycle‐to‐cycle and device‐to‐device variability for memory programming. For a typical GST‐*D*_250_ cell, we fixed the RESET voltage (*U*_RESET_) at 1.6 V and SET voltage at 1.2 V, with the pulse shape shown in the inset of Figure 3B,C. The electrical measurement of over 2 × 10^4^ switching cycles using this cell is presented in Figure 3D, showing consistently an ≈30‐fold contrast window between the average HRS and LRS values. This indicates that the crystal‐glass network pattern and the resulting conductive channels maintain similar morphology from cycle to cycle in the cycle range we experimented, because otherwise the resistance would have shown much larger variation. The high contrast stability also holds in our cell‐to‐cell uniformity tests, as shown in Figure 3E (we tested 30 independent GST‐*D*_250_ cells using similar operation protocols, and averaged the resistance of the first 100 cycles for each cell). Taking into account both cycling and inter‐device variations, the contrast in electrical resistance between the highest LRS and the lowest HRS is about fivefold, which is sufficient for binary storage. These results indicate that even though the network pattern reached through phase separation is unlikely to be identical for different memory cells, the functional conductive paths always bear sufficient similarity in physical dimensions and morphology, such that the performance of the resultant cells is satisfactorily reproducible, guaranteeing adequate reliability for binary programming applications.

The RESET energy can be estimated as *E*_RESET_ = *I* × *U* × Δ*t* = *U*_RESET_^2^ × Δ*t*/*R*_SET_. As shown in Figure 3D, the average *R*_SET_ upon cycling is ≈2.3 MΩ, and Δ*t* = 48 ns (full width at half maximum). The RESET energy is estimated as ≈53 fJ for this particular cell. After taking into account the minor cell‐to‐cell variations (e.g., slightly different *U*_RESET_ and *R*_SET_ for the 30 memory cells) shown in Figure 3E, the average RESET energy is found to be only ≈43 fJ. As presented in Figure 3F, all cells show consistently low power consumption at a few tens of fJ, always below that of CNT‐GST devices (80–100 fJ).^[^ ^34^, ^35^ ^]^ Note that if GST is used in the same *D*_250_ device setup, the average *E*_RESET_ value would be of nJ level,^[^ ^64^ ^]^ which is 4–5 orders of magnitude higher than the GSO‐*D*_250_ devices.

The low power consumption of GSO devices is further supported by the ultralow current needed for RESET operation. This is demonstrated using Figure S3, Supporting Information, in which a larger device, GSO‐*D*_500_, is directly programmed using current pulses. The pulse width was fixed as 103 ns (the minimum window of our home‐made testing setup), and the pulse amplitude was ramped up with 100 nA increments. RESET is accomplished at ≈1.5 µA, which is a current more than 3 orders of magnitude lower than that in GST‐*D*_250_ pore‐like devices, for which the current needed is as large as ≈3.0–11.4 mA.^[^ ^64^ ^]^ Notably, our *I*_RESET_ is comparable to that realized in nanowire devices, that is, the CNT‐GST devices (≈5 and ≈1.6 µA for CNT diameters of ≈3^[^ ^34^ ^]^ and ≈1.7 nm^[^ ^35^ ^]^). This is an indication that the effective switching volume for RESET operations in our device is indeed on nanoscale dimensions and the bridges being open and shut must be very few. What the RESET action does is only to break open the narrowest nano‐segment(s) of the conductive bridge, turning the cell into a HRS.

### 2.5 Cross‐Sectional Device Characterization Lending Support to the Switching Mechanism

**Figure 4:**
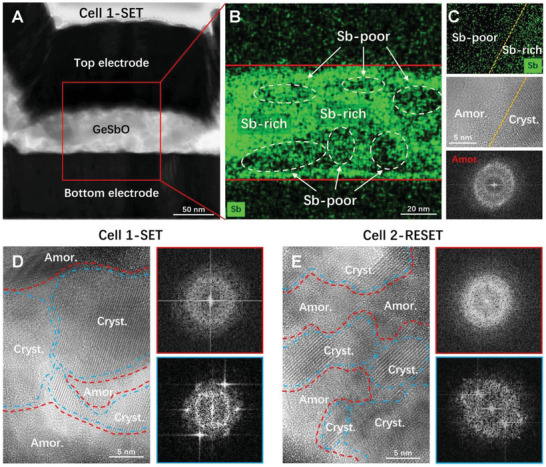
Cross‐sectional TEM showing intertwined nanodomains. A) An overview of a GSO‐*D*_250_ cell in SET state. The specimen is ≈80 nm in thickness along the viewing direction. B) The EDX element mapping of the middle part of the specimen. Sb‐poor and Sb‐rich domains are clearly identified. C) Local view of an interface area. The Sb‐poor domain shows maze‐like amorphous feature with a dim halo in the FFT pattern, while the Sb‐rich domain is clearly crystallized with lattice fringes. D) The high‐resolution TEM image and FFT of a local area in the cell in SET state, showing intermixed nanoscale crystalline and amorphous domains. The same also holds for RESET state: the networked mixture is observed in E) for another cell.

Next, we provide microstructural evidence lending support to the conductive bridge switching mechanism described above, using cross‐sectional TEM experiments on a couple of GSO‐*D*_250_ cells. **Figure** **4**A shows a slice of the device in SET state. The thickness along the viewing direction is ≈80 nm. Figure 4B shows a detailed map of Sb signals obtained using energy‐dispersive X‐ray spectroscopy (EDX), in which a clear variation, that is, undulating Sb‐rich and Sb‐poor regions, is observed. Figure 4C shows a local view that the Sb‐rich domain has a clear crystal lattice feature, while no crystalline feature is visible in the Sb‐poor domain. The dim halos in the FFT pattern also confirm the amorphous nature of the latter. We performed extensive high resolution TEM investigations of this SET state cell, and map out a complex network of amorphous and crystalline domains, as displayed in Figure 4D and Figure S5, Supporting Information.

These findings are consistent with what we predicted and discussed earlier, in regards to the microstructural features underlying the SET state. For the RESET state, we conducted similar experiments on a different cell. This memory cell also shows the expected network comprised of amorphous and crystalline domains with different chemical compositions, as shown in Figure 4E and Figure S6, Supporting Information. In other words, although it is not possible to know the exact location of the switching necks in such complex network and directly pinpoint the local phase transition that completed the conductive bridge in real devices, this set of observations in the unconfined TEM foils does serve the purpose of proving that Figure 1B is based on a correct microstructural picture. Again, our ultralow RESET current ≈1.5 µA, in comparison with the miniaturized nanowire CNT‐GST device with an ≈2.3 nm^2^ contact area, where the RESET current is ≈1.6 µA,^[^ ^35^ ^]^ is also a manifestation that the switching area involved in RESET operation must be on the scale of a few nm, despite the large (electrodes) contact area (≈10^5^–10^6^ nm^2^) used in our devices.

### 2.6 Defying the Scaling Between Switching Energy and Device Size

**Figure 5:**
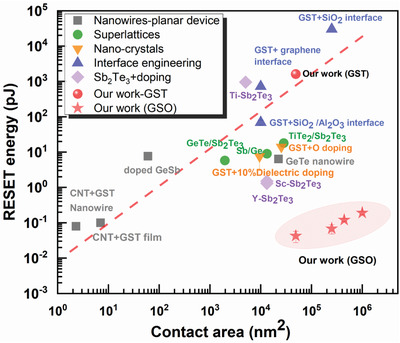
Power consumption in typical PCM devices. The RESET energy values are plotted with respect to the contact area with electrodes. The GSO‐*D*_250_ devices show an average *E*_RESET_ value of ≈43 fJ, which is more than 4 orders of magnitude lower than that of the GST‐*D*_250_ devices. The ultralow programming energy of our GSO‐based devices breaks the correlation relationship (dashed line) established for all previous devices, that is, the direct and proportional scaling of the power consumption with the size of the contact area with electrodes.

Finally, we examined the power consumption at larger device sizes. We tested multiple GSO‐*D*_500_ devices and always observed small *E*_RESET_ values below 100 fJ under suitable pulse setting. Only a slight increase to 150–200 fJ was found for even larger devices such as *D*_750_ and *D*_1000_. Such a low *E*_RESET_ suggests that the effective switching volume always remains small on nanoscale, further supporting the cbPCM switching mechanism. Moreover, as compared in **Figure** **5** with literature data plotted as a function of contact area size,^[^ ^12^, ^15^, ^20^, ^21^, ^22^, ^23^, ^24^, ^25^, ^26^, ^27^, ^28^, ^29^, ^30^, ^31^, ^32^, ^33^, ^34^, ^35^, ^50^, ^65^ ^]^ our cbPCM devices not only have achieved an unprecedented low power consumption in comparison with all devices of similar contact sizes, but also clearly breaks the previously established scaling (dashed line) of energy with the contact area. This is a major advantage of our heterogeneous alloy approach, as it achieves the minimization of the effective switching volume without the need to miniaturize the size of the electrodes or PCM cell, greatly reducing the complexity and costs for device fabrication. Before closing, we also note that the cbPCM switching mechanism is distinct from the conductive‐bridge mechanisms reported in filamentary resistive‐switching random access memories (RRAM) using metal oxides,^[^ ^3^, ^4^, ^5^ ^]^ as there the ionic conducting channels change their transport direction upon reversing the voltage polarity. In contrast, our cbPCM device shows unipolar switching feature (Figure S7, Supporting Information), confirming that phase transition is the underlying switching mechanism in our case. Such a phase transition mechanism occurring at certain local spots avoids the extensive ion transport as well as the randomness associated with the formation and dissolution of conductive filaments in RRAM.

## 3 Conclusion and Outlook

We have successfully designed and fabricated PCM devices with nanoscale conductive‐bridge using a carefully selected GSO alloy, breaking the energy‐size direct‐scaling correlation to reach a record low programming energy for phase‐change memory cells. To enable the new conductive bridge switching mechanism, we purposely “self‐patterned” the GSO microstructure to consist of a network with robust amorphous Sb‐poor domains intertwined with necklaces of Sb‐rich CNDs. The electric current meanders through a low‐resistance path, which connects many CNDs and forms a fully conductive path between the top and bottom electrodes, turning the memory cell into SET state. For RESET operation, the electric current flows along this conductive path and induces joule heating at narrower necks, which are surrounded by amorphous domains of higher electrical and thermal resistance. When these nanoscale necks turn amorphous, the conductive bridge is “shut,” rapidly increasing the overall cell resistance by at least one order of magnitude. The rapid switching speed (tens to hundreds of ns), and the minimized programming current (a few µA) together with the ultralow power consumption (tens of fJ), would enable 3D integration of PCM devices with many more stackable layers, and thus much increased data storage density. The alloy is compatible with the current semiconductor fabrication lines and can be achieved in one‐off sputtering, ensuring simple and low‐cost production. Scaling down the size of the device, but keeping it well above the amorphous/crystalline domain dimensions (5–20 nm), could further reduce the stochasticity of conductive channels as well as the device variation. Therefore, we advocate cbPCM as a potential candidate for phase‐change data storage in binary scheme. If needed, further optimization of the Ge‐Sb‐O chemical composition and potential doping is possible, to tailor the switching speed, thermal stability, as well as the size of the resistance window.

As a possible tweak toward yet another phase‐change application, our GSO‐based devices could also be made to present multi‐level resistance capability. To further widen the resistance window to accommodate more states, one could use stronger RESET pulses to enlarge the switching bridge or break more necks, or longer SET pulses to crystallize larger fractions of the heterogeneous network. This programming mode would however result in larger fluctuation and variability from cycle to cycle and from device to device, due to more extensive changes to the heterogeneous network. Nevertheless, such features might turn out to be useful for the emulation of stochastic phase‐change neurons,^[^ ^66^ ^]^ which play a key role in signal encoding and transmission in biological neural networks.^[^ ^67^ ^]^ With power consumption approaching biological neural network, the GSO‐based cbPCM devices may then enable performance superior to the conventional GST‐based PCM devices.

## 4 Experimental Section

### Film Synthesis and Characterization

Ge‐Sb‐O thin films were obtained by magnetron sputtering three alloy targets, Ge_7_Sb_93,_ Ge_10_Sb_90_, and Ge_15_Sb_85,_ under the same condition with a mixed argon and oxygen pressure of 0.5 Pa. The ≈100 nm thick Ge‐Sb‐O thin films were grown on SiO_2_/Si substrate and annealed at 200 °C for 30 min in a high vacuum furnace. The composition of the Ge_15_Sb_85_‐O thin film was identified as GSO using APT. For TEM characterization, the GSO films ≈80 nm in thickness were deposited onto ultra‐thin carbon film (≈5 nm) coated copper grids and were covered by ≈10 nm electron‐transparent ZnS‐SiO_2_ capping layers to prevent further oxidation. The samples were then annealed at 200 °C for 30 min in high vacuum furnace.

### Atom Probe Tomography Experiments

The needle‐shaped tip was prepared from the annealed GSO film by using the standard lift‐out process with a FEI Helios NanoLab 600 focus ion beam (FIB) system. The diameter of the tip was about 79 nm, and the tip was put into high vacuum (3 × 10^−11^ mbar) in a local electrode atom probe (LEAP 5000 XR, Cameca Instruments). By applying a DC voltage of 2.0–6.5 kV and illumination with 30 ps laser pulses in the APT chamber, atoms on the surface of the needle‐shape sample were ionized. These ions and ionic clusters were projected onto a position‐sensitive detector (PSD). The coordinates of ions or ionic clusters captured by the PSD were analyzed to reconstruct the 3D atomic map of the tip. The reconstruction and analyses of 3D maps were conducted using software IVAS 3.6.14.

### Transmission Electron Microscopy Experiments

The high‐resolution TEM and high‐angle annular dark‐field (HAADF) characterization of the GSO thin film samples were performed on a JEOL ARM200F microscope, operated at 200 keV with a low beam intensity. The recording time for TEM and HAADF images were a few seconds and ≈1 min, respectively. No visible structural changes were observed during the imaging process. Cross‐sectional TEM specimen of the GSO‐based devices were prepared using a FEI Helios 450 s dual beam FIB system with a Ga ion beam of 30 keV energy. The finishing touches for thinning and cleaning used lower energies of 5 and 2 keV, respectively. The final sample thickness was ≈80 nm. The high‐resolution TEM and energy dispersive X‐ray (EDX) spectroscopy characterizations were conducted at 200 kV on a FEI Titan Themis 200 microscope equipped with a spherical aberration corrector and a Bruker Super‐X EDX system. The TEM snapshots, EDX line scan, and EDX maps were performed using a low beam intensity within a few seconds, ≈2 min and ≈5 min, respectively.

### Device Fabrication and Electrical Measurements

Four Pt electrodes were patterned and sputtered on four corners of GSO films for in situ sheet resistance–temperature (*R*–*T*) measurement, which was performed using Keithley 4200 in a probe station of Instec mK2000 Temperature Controller with a heating rate of 6 °C min^−1^ in N_2_ environment. Pore‐like device structure was fabricated for electrical measurements. An ≈85 nm thick Pt layer was sputter‐deposited to serve as the bottom electrode. An ≈80 nm dielectric layer SiO_2_ coating was applied, via plasma‐enhanced chemical vapor deposition (PlasmaPro 800 Stratum), for heat diffusion inhibition and electrical insulation. The through holes (to fill in the GSO) were obtained through electron beam lithography pattering and SiO_2_ dry etching. Four different pores with the diameter ranging from 250 to 1000 nm were filled with ≈55 nm thick GSO film. Another 90 nm thick Pt film was deposited as the top electrode. The device performances were characterized using Keysight B1500A semiconductor device parameter analyzer, which could generate voltage pulses to stimulate the device. The resistance–current (*R–I*) measurements switched by electric current pulses were performed by a parameter analyzer (Keithley 2400C) and a current pulse generator (Tektronix AWG5200B).

### Ab Initio Simulations

DFT calculations were carried out with the Vienna Ab initio Simulation Package (VASP).^[^ ^68^ ^]^ The Perdew–Burke–Ernzerhof (PBE) functional^[^ ^69^ ^]^ and the projector augmented wave^[^ ^70^ ^]^ pseudopotentials were used for VASP calculations. The chemical bonding analysis were performed using the Local Orbital Basis Suite “Towards Electronic‐Structure Reconstruction” (LOBSTER) code.^[^ ^71^ ^]^ For the formation energy and chemical bonding analysis, the crystalline structures with the most favorable formation energy were chosen. DFMD were performed using the CP2K package^[^ ^72^ ^]^ and VASP. The PBE functional and the Goedecker pseudopotentials^[^ ^73^ ^]^ were used for CP2K calculations. A Ge‐Sb‐O model with 36 Ge, 288 Sb, and 36 O atoms was fully randomized at 3000 K for 10 ps and then quenched to 1000 K. The model was then quenched down to 0 K in 5 ps or 100 ps. For the slow‐quenched model, a snapshot was taken at 600 K, and Ge and O atoms were moved to the middle part and this segregated model was annealed at this temperature for 30 ps, then quenched down 0 K in 60 ps.

### Statistical Analysis

To analyze the device‐to‐device variation, resistance data in Figure 3E were obtained by switching 30 independent GSO cells, and each cell was operated over 100 cycles. The resistance values of each cell presented in Figure 3E were averaged over 100 cycles, with the error bars covering the mean ± standard deviation (SD) of the data. The statistic distribution of RESET energy in Figure 3F was calculated from the mean resistance value in Figure 3E. The RESET energy of the GSO devices in Figure 5 was averaged over all GSO cells of the same size, with the error barspanning across the mean ± SD of the RESET energy data.

## Conflict of Interest

The authors declare no conflict of interest.

## Supporting information

Supporting Information

## Data Availability

All data are available in the manuscript or the Supporting Information, and are available from the corresponding authors upon reasonable requests.
