# Supplementary material for: Designing Conductive‐Bridge Phase‐Change Memory to Enable Ultralow Programming Power
Source: Adv Sci (Weinh). 2022 Jan 14;9(8):2103478. doi: 10.1002/advs.202103478 (PMC8922100; doi:10.1002/advs.202103478)
Supplement: Supplementary file 1 — Supporting Information [file ADVS-9-2103478-s001.pdf]

Supporting Information

for *Adv. Sci.,* DOI: 10.1002/advs.202103478

Designing conductive-bridge phase-change memory to enable ultralow programming power

Zhe Yang, Bowen Li, Jiang-Jing Wang, Xu-Dong Wang, Meng Xu, Hao Tong, Xiaomin Cheng, Lu Lu, Chunlin Jia, Ming Xu, Xiangshui Miao,* Wei Zhang,* and En Ma**

Supporting Information

Designing conductive-bridge phase-change memory to enable ultralow programming power

Zhe Yang, Bowen Li, Jiang-Jing Wang, Xu-Dong Wang, Meng Xu, Hao Tong, Xiaomin Cheng, Lu Lu, Chunlin Jia, Ming Xu, Xiangshui Miao, Wei Zhang, and En Ma

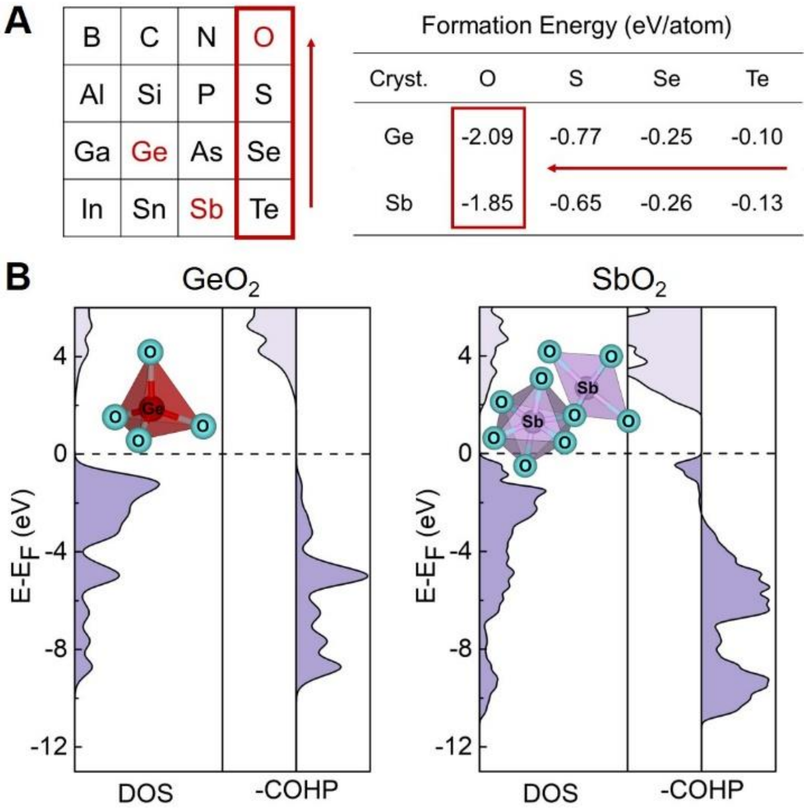

Figure S1. Formation energy and chemical bonding analysis of crystalline GeO_2 and SbO_2. (**A**) Formation energy for Ge- and Sb- oxides and chalcogenide crystals with most favorable formation energy – GeO_2 ($P3_121$), GeS_2 ($Fdd2$), $GeSe_2$ ($I\bar{4}2d$), GeTe ($R3m$), SbO_2 ($Pna2_1$), Sb_2S_3 ($Pnma$), Sb_2Se_3 ($Pnma$) and Sb_2Te_3 ($R\bar{3}m$). (**B**) Chemical bonding analyses for GeO_2 and SbO_2. Insets in (**B**) are the structural motifs of the corresponding compounds. The left and right parts of the -COHP curves indicate antibonding (destabilizing) and bonding (stabilizing) interactions, respectively. GeO_2 shows all bonding interactions below the Fermi level, while there exhibits a small antibonding interaction in SbO_2 below the Fermi level, indicating some chemical instability.

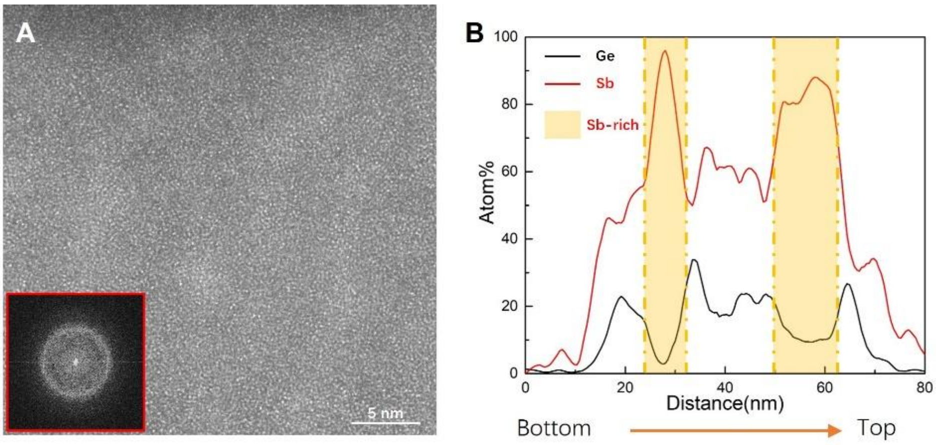

Figure S2 Cross-sectional TEM characterization and elemental analyses of an as-fabricated device cell. (**A**) The HRTEM image and corresponding fast Fourier transform (FFT) pattern. The diffuse halo in FFT pattern indicates that the alloy is fully amorphous. (**B**) The EDX line-scan of the as-fabricated cell from bottom electrode to top electrode shows clear chemical separation, i.e., compositional segregation inside the amorphous phase.

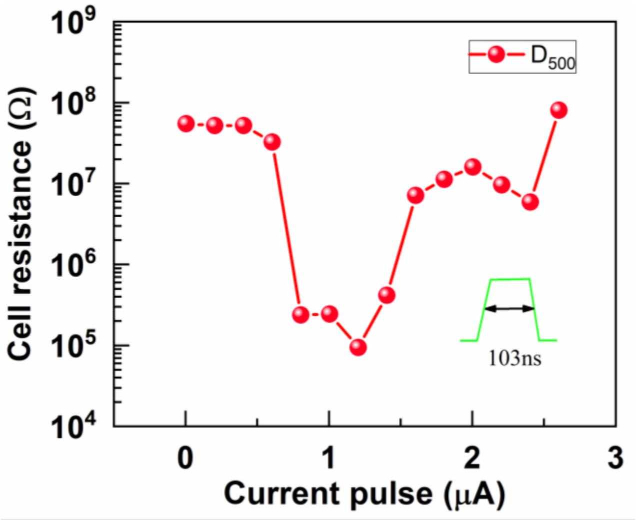

Figure S3. GSO-D_{500} device operation driven by current pulses. Cell resistance versus current amplitude. A sharp decrease and increase of resistance is observed at ~0.5 μA and ~1.5 μA, corresponding to SET and RESET operation, respectively. The pulse width is set to be 103 ns including 3 ns falling edge. The current required for switching is expected to be further reduced in the smaller GSO-D_{250} devices.

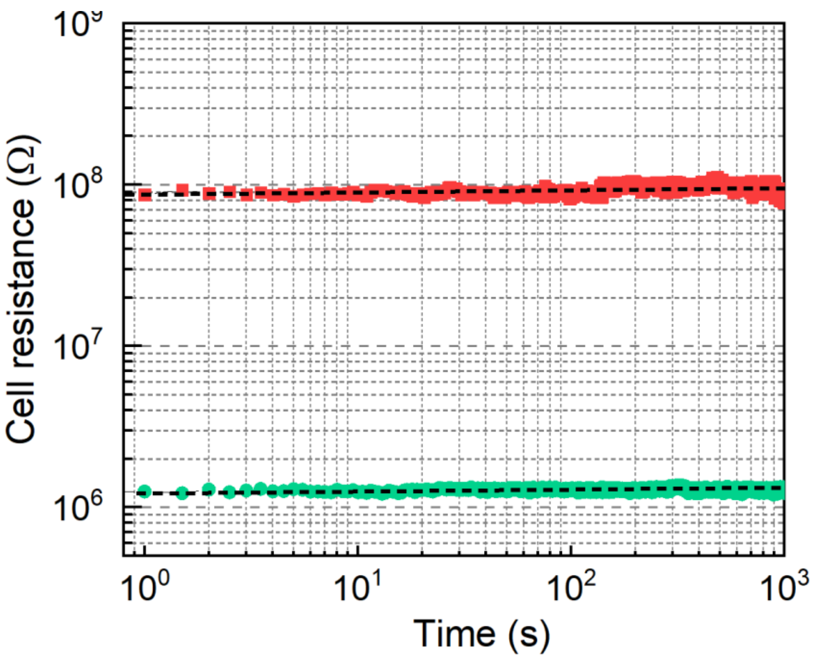

Figure S4 Drift measurement at room temperature. A GSO-D250 memory cell is programmed into HRS and LRS for resistance measurement over time. The applied voltage is 10 mV. The measured resistance curves obey the power law, R (t) = R_0 $(t/t_0)^{v}$. R_0 is the initial resistance at time t_0. The fitted drift coefficient v is ~0.003 for LRS (green) and ~0.014 for HRS (red).

Cell 1-SET

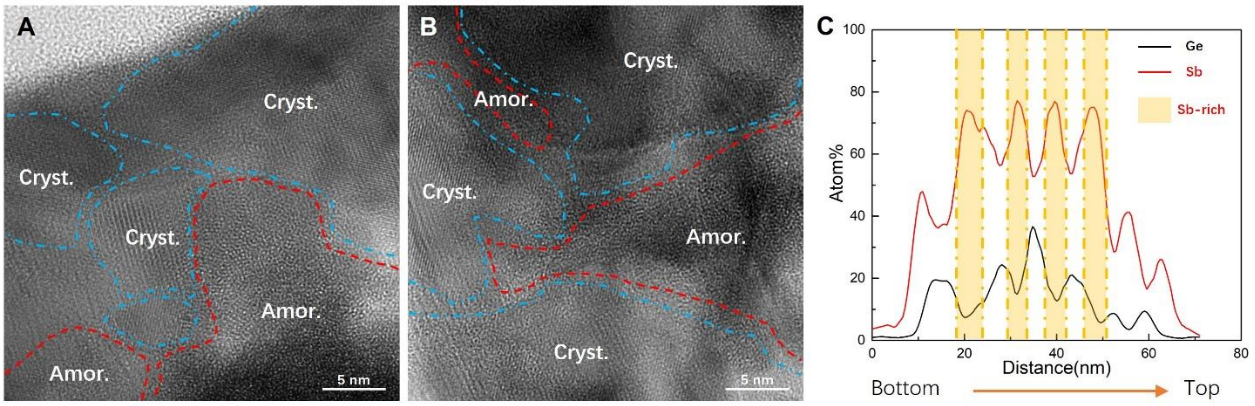

Figure S5. Cross-sectional TEM characterization and chemical mapping of a cell in SET state. (**A**) and (**B**) The HRTEM images of a SET cell. The amorphous and crystalline domains are encircled using red and blue dashed lines, respectively, confirming the intertwined network of amorphous and crystalline nanodomains. (**C**) The EDX line-scan of the RESET cell from bottom electrode to top electrode clearly demonstrates the compositional variation on nanoscale.

Cell 2-RESET

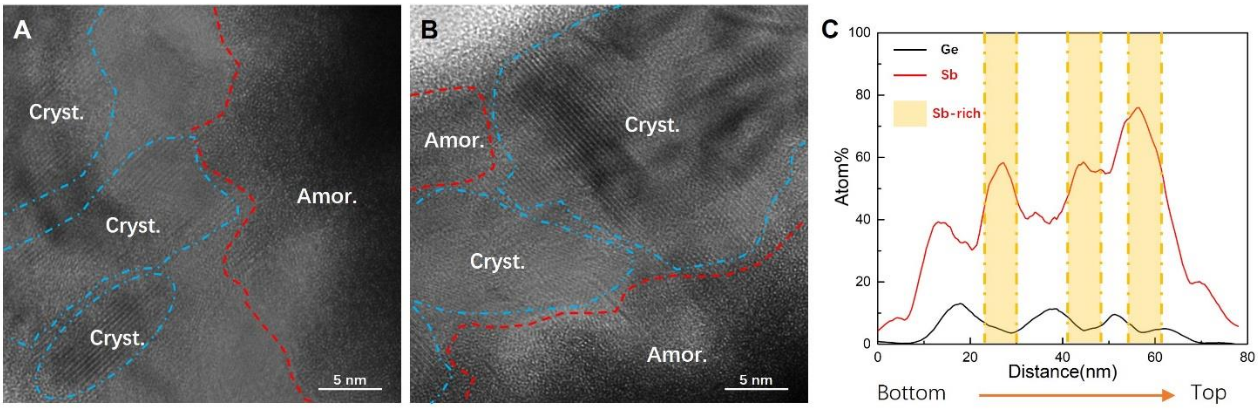

Figure S6. Cross-sectional TEM characterization and chemical mapping of a cell in RESET state. (**A**) and (**B**) The HRTEM images of a RESET cell. The amorphous and crystalline domains are encircled using red and blue dashed lines, respectively, confirming the intertwined network of amorphous and crystalline nanodomains. (**C**) The EDX line-scan of the RESET cell from bottom electrode to top electrode clearly demonstrates the compositional variation on nanoscale.

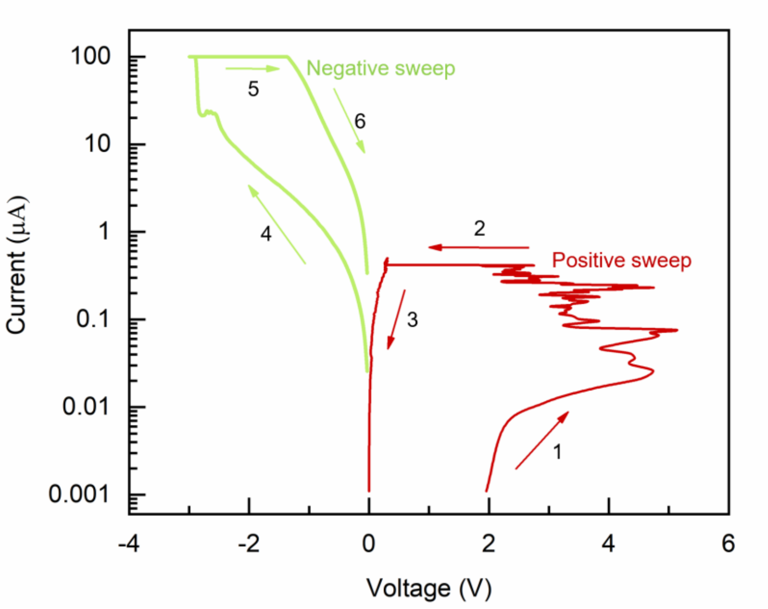

Figure S7 Bidirectional DC sweeps on a cbPCM device. The positive sweep (red curve) establishes the conductive necklace in the initial as-deposited sample, setting the device from HRS to LRS. The negative sweep (green curve) could further set the device into an even lower resistance. Such a unipolar feature of our device demonstrates that the cbPCM is driven by phase transition, rather than ion diffusion in which the negative sweep would reset the device back into HRS.
